# An Embedded-Sensor Approach for Concrete Resistivity Measurement in On-Site Corrosion Monitoring: Cell Constants Determination

**DOI:** 10.3390/s21072481

**Published:** 2021-04-02

**Authors:** Jose Enrique Ramón, Isabel Martínez, José Manuel Gandía-Romero, Juan Soto

**Affiliations:** 1 Instituto de Ciencias de la Construcción Eduardo Torroja, CSIC, C/Serrano Galvache No.4, 28033 Madrid, Spain; isabel.martinez@csic.es; 2 Department of Architectural Construction, Universitat Politècnica de València, Camino de Vera s/n, 46022 Valencia, Spain; joganro@csa.upv.es; 3 Interuniversity Research Institute for Molecular Recognition and Technological Development, Universitat Politècnica de València—Universitat de València, Camino de Vera s/n, 46022 Valencia, Spain; juansoto@qim.upv.es

**Keywords:** structure health monitoring, resistivity, sensor, non-destructive technique, steel corrosion, reinforced concrete, durability

## Abstract

The concrete electrical resistivity is a prominent parameter in structural health monitoring, since, along with corrosion potential, it provides relevant qualitative diagnosis of the reinforcement corrosion. This study proposes a simple expression to reliable determine resistivity from the concrete electrical resistance (R_E_) provided by the corrosion sensor of the Integrated Network of Sensors for Smart Corrosion Monitoring (INESSCOM) we have developed. The novelty here is that distinct from common resistivity sensors, the cell constants obtained by the proposed expression are intended to be valid for any sensor implementation scenario. This was ensured by studying most significant geometrical features of the sensor in a wide set of calibration solutions. This embedded-sensor approach is intended to be applicable for R_E_ measurements obtained both using potential step voltammetry (PSV, used in the INESSCOM sensor for corrosion rate measurement) and alternating current methods. In this regard, we present a simple protocol to reliably determine R_E_, and therefore resistivity, from PSV measurements. It consists in adding a very short potentiostatic pulse to the original technique. In this way, we are able to easy monitor resistivity along with corrosion rate through a single sensor, an advantage which is not usual in structural health monitoring.

## 1. Introduction

The service life of reinforced concrete structures depends largely on the extent of reinforcement corrosion, known to begin when certain aggressive agents such as CO_2_ and chloride ions come into contact with and destroy the passive film formed on the steel rebars [1]. Hence the importance of concrete cover quality, for it is the sole physical-chemical barrier between the steel and the exposure environment. Corrosion rate depends on ionic transport in the concrete between the anode and cathode of the electrolytic cells forming in the reinforcement [2]. Concrete physical-chemical properties therefore determine the corrosion rate. Whilst durability is an essential item in today’s standards on concrete, it is not unusual in practice to find structures with poor quality cover concrete usually due to an inadequate control of the curing conditions [3].

For all this, concrete cover resistivity is an important parameter to take into account to study reinforcement durability, as it provides information about the concrete quality and its saturation degree, directly related with the corrosion process [4]. Several authors have revealed an inversely proportional relationship between concrete resistivity and reinforcement corrosion rate [5,6]. Resistivity, along with corrosion potential, is consequently a prominent parameter in most inspection and monitoring systems [7]. Although qualitative, it is a parameter useful for detecting areas with potential risk of corrosion. Recommendations for correlating the probable corrosion risk and concrete resistivity are presented in the literature [8], where the corrosion risk of reinforcement is divided into four levels according to the magnitude of resistivity (ρ) as exemplified at Table 1 together with corrosion risk criteria based on the corrosion rate value, expressed in terms of current density (i_CORR_), according to references [9,10].

The Wenner (four electrodes) and the disc (one external electrode) methods are the benchmark techniques for in situ resistivity measurements in existing concrete structures [8]. Wickramanayake et al. [11] and Feliu et al. [12] developed outstanding sensors which operate on these respective principles. Given the utility of broaching durability problems effectively and sufficiently in advance, however, by monitoring structures from the time they are built, alternative embedded methods for monitoring resistivity have been proposed. Examples include a multi-electrode system developed by Badr et al. [13] to determine resistivity at different depths or the screen-printed sensor proposed by Sophocleous et al. [14]. Inasmuch as resistivity is particularly helpful when analyzed in conjunction with other durability parameters, the idea of integrating resistivity measurements in embedded corrosion rate assessment systems has been gaining in popularity [15,16]. In this line, Duffó et al. [17] proposed a multi-parameter sensor in which electrical resistance measured between two inert electrodes is converted to resistivity by applying a cell constant. Common commercial devices are based on this principle to determine resistivity in corrosion monitoring [18], though there are some examples that use Wenner-based sensors [19].

The need to incorporate a specific resistivity sensor into the monitoring system could involve increasing the implementation costs and complexity of the system. In addition, under certain circumstances, some resistivity sensors could reduce their reliability because the cell constants used are usually limited to very specific implementation conditions [8]. It is usually due to the use of a limited number of calibration solutions [13], as well as due to variations in the geometric features of the sensor during its implementation which have not been considered in the cell constant calibration [10]. Therefore, it is usually suggested to calibrate the cell constant in every sensor implementation to ensure reliability [10,20].

Technological advances in recent decades have also influenced the development of corrosion sensors. Among the most important proposals are fiber optic sensors [21,22], which stand out for their versatility and miniaturization capability; and sensors based on inductively coupled magnetic fields [23,24], which allow wireless monitoring. In addition, emerging damage identification techniques based on acoustic emission [25] and guided ultrasonic waves [26] have proved effective in damage identification in reinforced concrete. Despite their unquestionable advantages, at the moment these advanced methods do not provide high accuracy in corrosion rate measurements and, something which take special importance in the context of this paper, do not include the possibility of determining the electrical resistivity of concrete.

The present authors have been studying the durability issues arising around reinforced concrete for many years, focusing particularly on modelling and developing methods for corrosion rate determination [27,28,29]. The outcome is an innovative system of embedded sensors for monitoring corrosion in reinforced concrete structures [30,31], which we have termed INESSCOM (an acronym for Integrated Network of Sensors for Smart Corrosion Monitoring). This autonomous system can monitor several areas of a structure simultaneously and in real time using an embedded sensor in which an innovative approach with potential step voltammetry (PSV) provides a number of parameters, including corrosion rate and electrical resistance of concrete [29], but not electrical resistivity, which could limit its applicability.

Although the authors have discussed and put forward alternatives for measuring resistivity in earlier articles, those studies were geared towards the resistivity determination in laboratory studies [32]. Whilst the conclusions of those prior efforts are useful, the present study is geared toward a different perspective, namely on-site concrete resistivity determination.

The primary aim of this work was to define a reliable expression to determine resistivity from the concrete electrical resistance (R_E_) provided by the INESSCOM corrosion sensor. The novelty here is that distinct from common embedded sensors, the constant cells of the proposed expression are applicable for any sensor implementation scenario. This was ensured by studying most significant geometrical features of the sensor, i.e., electrode areas, spacing and positions/setups, by using a wide set of calibration solutions which quite cover the range of values that would be expected in on-site resistivity monitoring.

This proposal was designed to be to be applicable for R_E_ measurements obtained both using the PSV method, implemented in the INESSCOM sensor for corrosion rate measurement, and alternating current methods. In this regard, as an added novelty, we present a simple PSV protocol to reliably determine R_E_, and therefore resistivity, by using the INESSCOM corrosion sensor. This renders feasible to quick and easy monitor resistivity along with corrosion rate through a single sensor, thus eliminating the need for additional sensors, time-consuming methods or complex electronic devices; an unusual advantage in structural health monitoring.

## 2. Materials and Methods

### 2.1. Measurement Principle Governing INESSCOM Sensor

**Figure 1:**
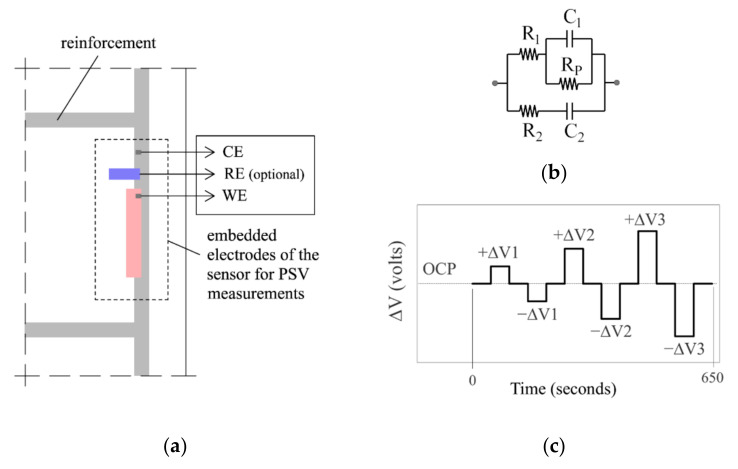
Embedded corrosion sensor for measuring the corrosion rate: (**a**) measuring cell used to apply PSV with the optional inclusion of a reference electrode (RE) to measure the E_CORR_; (**b**) equivalent circuit used in PSV to model the transient response of the steel-concrete system; and (**c**) potential pulse sequence applied in PSV to polarize the system.

Figure 1a depicts the corrosion measurement principle governing the INESSCOM sensor we have developed and whose possible use to determine concrete resistivity is explored hereunder. The system uses potential step voltammetry (PSV) to measure corrosion rate, as described elsewhere [29]. The technique is based on the intersection method, with the added advantage that as the Tafel slopes can be found in less time, the technique is less invasive than the original method. The circuit in Figure 1b proposed in earlier studies [27] is used to model the transient response of the steel-concrete system when the potential pulse sequence shown in Figure 1c was applied. In addition to corrosion rate, the model can be used to find parameters such as the electrical resistance of concrete (R_E_-_PSV_), based on circuit resistances R_1_ and R_2_ from Equation (1):

(1) $$R_{E-\mathrm{PSV}}=\frac{R_{1}\cdot R_{2}}{R_{1}+R_{2}}$$

The sensor needs only two elements (Figure 1a) for conducting the PSV measurement, the working (WE) and counter (CE) electrodes. The advantage of such a two-electrode setup is that, unlike standard sensors, it does not require an embedded reference electrode (RE) whose uncertain long-term stability could affect sensor reliability. The sensor may, however, optionally bear a built-in RE to measure the corrosion potential (E_CORR_) which, although a qualitative parameter, provides more complete analyses of reinforcement condition.

In the two-electrode PSV measurements (Figure 1a), the WE was a corrugated carbon steel bar with the same characteristics as the reinforcement of the structure to be monitored, but shorter and with the two ends sealed with epoxy resin to clearly delimit the working area. The CE, in turn, was the reinforcement itself of the structure to be monitored. The CE area was consequently much larger than the WE area, an arrangement shown in earlier studies [31] to be imperative for reliable two-electrode PSV measurement.

### 2.2. Measuring Cell

**Figure 2:**
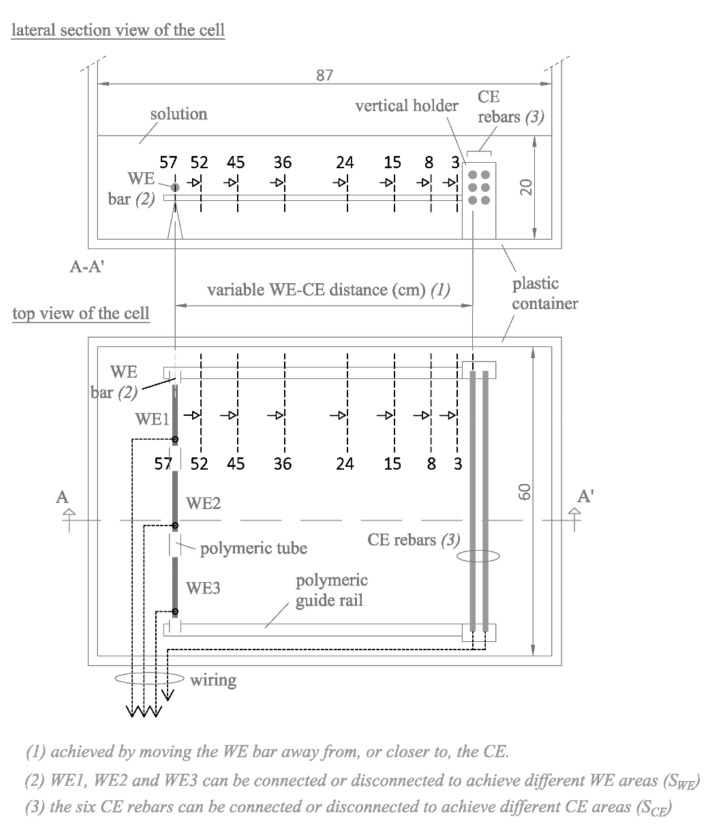
Measuring cell used to assess the effect of the S_CE_/S_WE_ ratio and WE-CE spacing (dimensions in cm).

**Figure 3:**
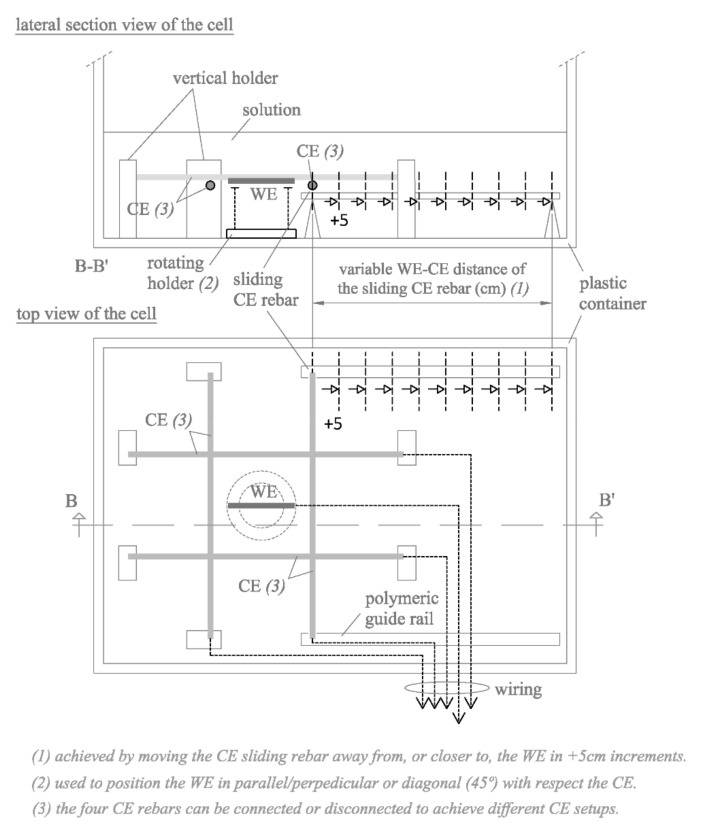
Measuring cell used to assess the effect of WE position relative to the rebars in the CE and the effect in the case that one of the CE rebars is placed at a different WE-CE distance with respect to all the others (dimensions in cm).

Cells were designed to measure electrical resistance (R_E_) between one or several working electrodes (WE) and the counter electrode (CE) housed in the corrosion sensor described in Section 2.1. Two types of cell were used (Figure 2 and Figure 3) to assess the maximum number of possible geometrical factors that may be relevant to R_E_ conversion to resistivity (ρ).

The cell in Figure 2 was intended to assess how the CE area/WE area (S_CE_/S_WE_) ratio affected the sensor. Both the WE and the CE comprised several rebars that could be connected or disconnected as needed, thereby generating a wide spectrum of S_CE_/S_WE_ ratios. In the CE, six rebars were positioned in a polymeric holder in three two-column rows and evenly spaced at 1.5 cm. The three WE (WE1, WE2 and WE3) were arranged as a single linear rebar using polymeric tubes and it was mounted on polymeric guide rails to be placed parallel at variable distances to the CE. With that setup, the cell could be used to assess the effect of the WE-CE spacing on measurements, for which purpose eight WE-CE distances were studied: 3, 8, 15, 24, 36, 45, 52 and 57 cm. This wide range of distances is intended to provide sufficient variation of the WE-CE spacing and, thus, to reliably calibrate the cell constant according this important geometrical factor of the sensor. Moreover, these WE-CE distances are in the range of usual spacing of rebars in concrete structures [33], so they can be considered realistic.

The cell in Figure 3 consisted in a four-rebar lattice CE, whose rebars could be connected or disconnected at will to establish different CE setups, and a single-rebar WE positioned on a rotating holder to adjust its relative position to the CE.

**Figure 4:**
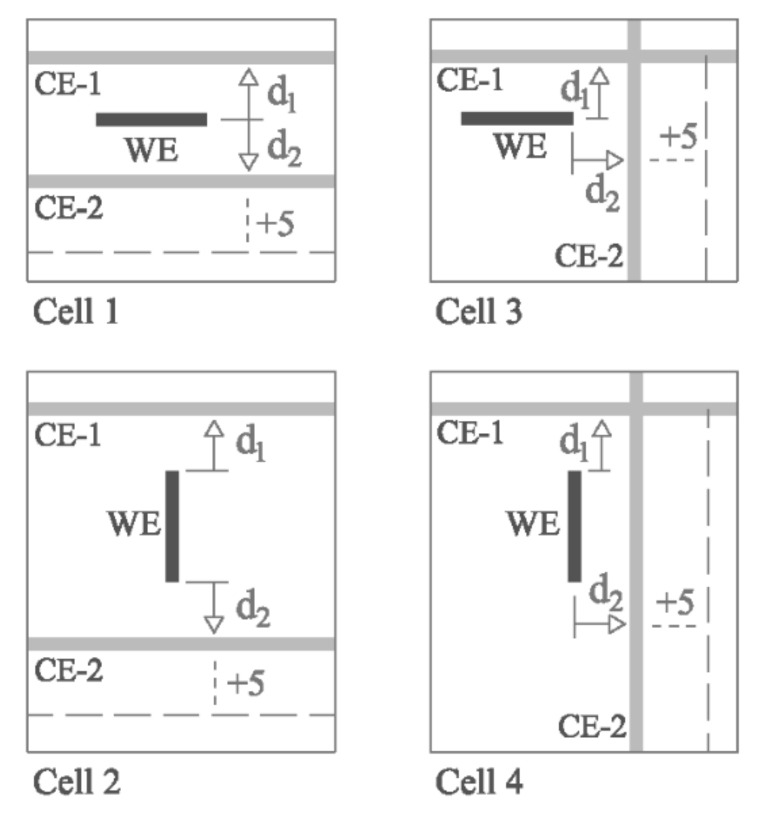
Four cell arrangements (from the cell in Figure 3) used to study the effect of unequal distances between the CE bars and the WE. The CE consisted in two rebars, CE-1, placed at a constant 5 cm (d_1_) from the WE and CE-2, positioned at variable distances (d_2_) with upward ramping at 5 cm intervals.

On the one hand, the cell in Figure 3 led to the four cell arrangements shown in Figure 4, which were used to study the effect in the case that one of the CE rebars (CE-2) is placed at a different WE-CE distance (d_2_) with respect to a second CE rebar (CE-1) at a constant distance (d_1_) from the WE. To do this, one of the CE rebars was mounted on lateral guide rails to be slid in 5 cm steps to progressively increase its WE-CE distance (d_2_).

**Figure 5:**
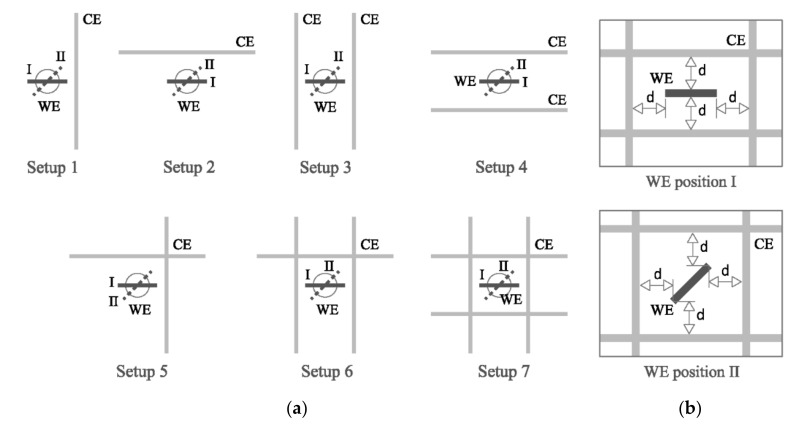
(**a**) Seven WE-CE setups (from the cell in Figure 3) used to assess the effect of the position of WE relative to CE on resistivity measurements; (**b**) WE positions relative to CE in the WE-CE setups: (I) parallel/perpendicular or (II) diagonal (45°).

On the other hand, the cell in Figure 3 led to the seven WE-CE setups shown in Figure 5a, which were used to analyze how the WE position with respect the CE (arranged in various manners) affected measurements. Each setup was considered to have two possible WE orientations relative to the CE as shown in Figure 5b: (I) in parallel/perpendicular or (II) in diagonal (45°). Here, all four CE rebars were at a constant 5 cm from the WE.

All the WEs employed consisted of a corrugated carbon steel bar of 8 mm in diameter and 70 mm in length sealed at both ends by pouring epoxy resin into a polymeric tube to delimit the working area and protect the electrical connection to the copper wire installed at one of the ends. The effective working area for each WE element was 17.5 cm^2^. The CE was made of the same steel as the WEs. Each CE rebar consisted in a rebar of 8 mm in diameter and 420 mm in length wired and sealed in the same way as the WEs. The working area of each CE rebar was 105.6 cm^2^.

### 2.3. Solutions

Measurements were taken in 15 aqueous solutions ranging in resistivity from 6.5 to 65,500 Ω·cm. This wide range of values is intended to cover as many different implementations scenarios as possible in the sensor characterization. The main field of application of the sensor is for concrete monitoring. In the case of ordinary concretes, resistivity may vary, according to [8], from 10,000 to 100,000 Ω·cm. However, less resistive concretes are also of interest, such as steel fiber [34] and carbon fiber [35] reinforced concrete.

The composition, pH and resistivity of the solutions used are listed in Table 2. Three types of solvent were used, A, B and C. Five solutions were prepared with solvent A, a saturated Ca(OH)_2_-tap water solution (with 0.0005 mol·L^−1^ of chlorides) to simulate the solution in concrete pores. Seven solutions were based on solvent B, tap water alone in which the absence of Ca(OH)_2_ determined higher resistivity, closer to the values characteristic of concrete. Inasmuch as solvent C consisted in deionized rather than tap water, the three resulting solutions exhibited the highest resistivity used in this study. NaCl was added at different concentrations in each set of solutions (Table 2) to vary resistivity across a broader range of values. Solution A5 (saturated Ca(OH)_2_ with 1 mol·L^−1^ chloride) simulated concrete carbonation by adding NaHCO_3_, which lowered the pH to 8.8. All the solutions were prepared with analytical grade reagents.

The study was performed from lower to higher chloride concentration, adding the respective amount of NaCl at each step, thereby avoiding the need to prepare a new solution for each chloride concentration in the set. Otherwise, the experiment would have been resource-intensive, for measurements were made in an 870 × 600 × 510 mm^3^ plastic container filled to a height of 200 mm, yielding a volume of 104.4 L of solution (Figure 2 and Figure 3).

### 2.4. Measuring Procedure

The WE and CE were degreased with acetone, washed in alcohol and hot-air dried prior to measuring. No surface treatment was applied after soaking to reproduce the conditions actually prevailing in the use of the sensor system in concrete. All experiments were conducted at laboratory temperature (20 ± 2 °C). The WE and CE were exposed to each volume-verified solution (Table 2) for 12 h, deemed sufficient to attain a stationary potential. Solution resistivity and temperature were subsequently measured on a Hanna Instruments HI 2300 auto-ranging resistivity meter, after which sensor measurements were undertaken.

The first parameter measured was electrical resistance (R_E_) between the WE and CE in all the cell types and combinations in Figure 2 and Figure 3, as described in Section 2.2, using a U1733C handheld alternating current (a.c.) LCR meter (Keysight Technologies, Santa Rosa, CA, USA). This R_E_ measurement was contrasted with the R_E_ value obtained by PSV technique as described in Section 2.1 for all the WE-CE distances depicted in Figure 2. The corrosion current density (i_CORR_) was also obtained from the PSV measurement as described elsewhere [29]. The potential step sequence applied in the PSV measurements was as shown in Figure 1c, with ±∆V1 = 105 mV, ±∆V2 = 140 mV and ±∆V3 = 175 mV relative to the open circuit potential (OCP). The R_E_ value found was compared to the resistance determined with a 50 mV, 5 ms pulse. That fast pulse was applied to obtain a more reliable sensor response at the outset of the test (t → 0) when the non-Faraday processes that determine R_E_ prevail. The PSV measurements were taken on a PGSTAT 100 potentiostat (Metrohm Autolab, Utrecht, The Netherlands). The OCP used was deemed in all cases to be the potential recorded between the WE and CE when the ∂E/∂t value dropped to below 0.03 mV/s.

## 3. Results

This study explored a simple procedure for determining resistivity (ρ) with the INESSCOM embedded sensor described in Section 2.1. It is designed to measure corrosion rate in a two-electrode (WE and CE) setup. Equation (2), used in the two-point or direct method where the resistivity measurement is also taken between two electrodes, was consequently adopted as the initial reference:

(2) $$\rho =R_{E}\cdot \frac{S}{d}$$

Equation (2) determines resistivity (ρ) in a concrete sample with length d and cross-section S from the electrical resistance (R_E_) measured between two equal flat electrodes, likewise with cross-section S and positioned in parallel on opposite sides of the sample.

The two electrodes in the corrosion sensor used here, however, were not flat, had different cross-sections and could not be positioned in parallel. In addition, the sensor measurement is based on the Potential Step Voltammetry (PSV), whereas common methods for resistivity measurement are based on alternating current methods.

Based on these factors, and to facilitate the analysis and discussion of the results, this section has been divided into two main phases:

(1) Study of the geometrical cell parameters in sensor
  Here we study the different geometrical factors involved in the sensor measuring cell which could affect resistivity determination. Each factor has been varied enough to determine the most appropriate manner to be incorporated into Equation (2), i.e., as a cell constant, in order to obtain a reliable expression to determine resistivity. In consequence, this phase has been divided in different sub-sections, each one focused on a specific feature of the sensor:
  (i) Electrode areas,
  (ii) Electrode spacing and
  (iii) Electrode arrangement.
(2) Reliability of the PSV-measured RE
  Here we analyze if the R_E_ obtained by the PSV method (used in the sensor for corrosion rate measurement) can be directly introduced in the expression determined in the previous stage to determine resistivity or whether a feasible PSV modification is required to ensure accuracy.

### 3.1. Study of the Geometrical Cell Parameters in Sensor

#### 3.1.1. Electrode Areas

**Figure 6:**
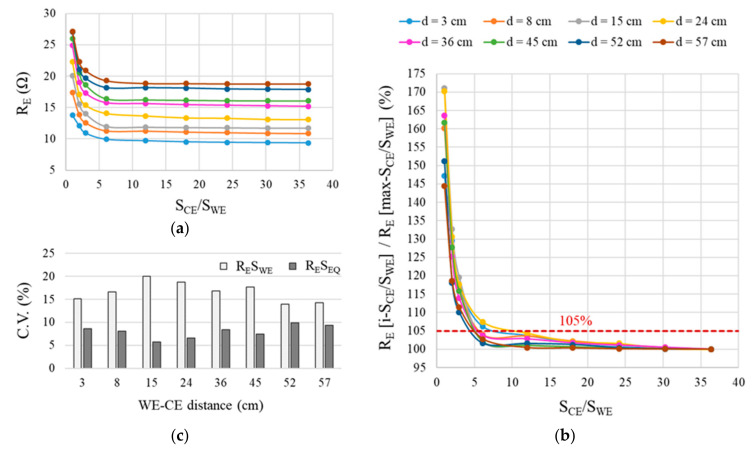
Electrical resistance (R_E_) at different WE-CE distances in a saturated Ca(OH)_2_ solution with 0 mol·L^−1^ chlorides: (**a**) R_E_ vs. the S_CE_/S_WE_ ratio; (**b**) R_E_[i-S_CE_/S_WE_]/R_E_[max-S_CE_/S_WE_] vs. S_CE_/S_WE_ where R_E_[i-S_CE_/S_WE_] is the R_E_ measured at the respective i-S_CE_/S_WE_ and R_E_[max-S_CE_/S_WE_] is the R_E_ obtained for the maximum S_CE_/S_WE_; and (**c**) coefficient of variation in R_E_·S depending on whether S is defined as S_WE_ or S_EQ_.

Electrical resistance (R_E_) was measured with the cell in Figure 2, varying the number of bars connected to the CE and to the WE. That procedure yielded measurements for nine CE area/WE area (S_CE_/S_WE_) ratios for each of the eight possible distances between the WE and CE. The findings for the Ca(OH)_2_-saturated solution with 0 mol·L^−1^ of chlorides (solution A1 in Table 2) plotted in Figure 6a show that R_E_ values tended to stabilize at sufficiently large S_CE_/S_WE_ ratios. Figure 6b graphs the relationship between R_E_ for each S_CE_/S_WE_ ratio (termed as R_E_[i-S_CE_/S_WE_]) and the R_E_ for the maximum S_CE_/S_WE_ ratio (termed as R_E_[max-S_CE_/S_WE_]). The difference in the measurements was <5% when CE was 12 times greater than WE, as was the case at all the distances studied.

The S_CE_/S_WE_ threshold of >12 would be guaranteed if sensors are used to monitor reinforced concrete structures, for in such cases, as noted in Section 2.1, the CE is the reinforcement itself. It would nonetheless be useful to be able to apply resistivity for any possible S_CE_/S_WE_ ratio. It is not unusual in research, for instance, to monitor small or lightly reinforced concrete specimens, where the sensor’s S_CE_/S_WE_ ratio is lower.

A method for normalizing R_E_ for any S_CE_/S_WE_ ratio was consequently needed. In Equation (2) the R_E_ value is multiplied by the sensor’s working area (S). The resulting coefficient of variation (C.V.) for each WE-CE distance is shown in Figure 6c. The findings showed that the C.V. for R_E_S_WE_ was over 15% at WE-CE distances of 3 cm to 45 cm and from 10% to 15% at distances of 52 cm to 57 cm. The C.V. declined to under 10% for R_E_S_EQ_, however. The parameter S_EQ_, proposed to express the sensor’s equivalent working area, is defined in terms of S_CE_ and S_WE_ as follows:

(3) $$S_{\mathrm{EQ}}=\frac{S_{\mathrm{WE}}\cdot S_{\mathrm{CE}}}{S_{\mathrm{WE}}+S_{\mathrm{CE}}}$$

Another factor that must be taken into consideration in Equation (3) is that the rebars comprising the CE may not all be at the same distance from the WE. Therefore, R_E_ was measured between the WE and a CE consisting of two bars (CE-1 and CE-2), one at a constant 5 cm (d_1_) from the WE and the other at d_2_, which varied. That yielded a number of d_2_/d_1_ ratios for all four cell arrangements depicted in Figure 4.

**Figure 7:**
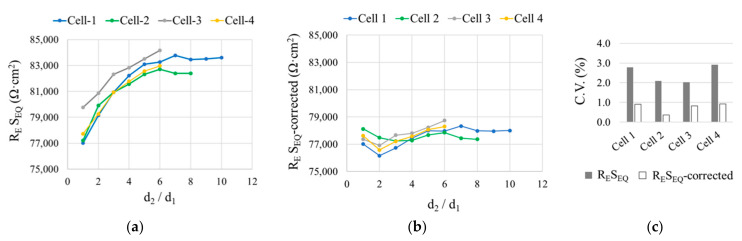
(**a**) R_E_S_EQ_ vs d_2_/d_1_ for a two-rebar CE (CE-1 and CE-2), one at a fixed (d_1_) and the other at a variable (d_2_) WE-CE distance, using the four cells in Figure 4 in a deionized water solution with 0 mol·L^−1^ of chlorides. (**b**) R_E_S_EQ_-corrected vs d_2_/d_1_. S_EQ_-corrected results by using S_CE_EQ_ (Equation (5)) to calculate S_EQ_ (Equation (4)). (**c**) Coefficient of variation (C.V.) for R_E_S_EQ_ and R_E_S_EQ_-corrected at all the d_2_/d_1_ ratios.

As Figure 7a shows, here S_EQ_ as defined in Equation (3) would not be suitable, since the R_E_S_EQ_ value varied upward with rising d_2_/d_1_ ratios in all four setups. To correct for that effect, the areas of CE-1 and CE-2 had to be used in S_EQ_ calculations in proportion to their respective distance from the WE. The proposal consequently consisted in replacing the term S_CE_ in Equation (3) with S_CE_EQ_, as follows:

(4) $$S_{\mathrm{EQ}}=\frac{S_{\mathrm{WE}}\cdot S_{\mathrm{CE}\_\mathrm{EQ}}}{S_{\mathrm{WE}}+S_{\mathrm{CE}\_\mathrm{EQ}}}$$

S_CE_EQ_ is found as the sum of the equivalent areas of all the rebars comprising the CE:

(5) $$S_{\mathrm{CE}\_\mathrm{EQ}}=\overset{n}{\underset{i=1}{\sum }}S_{\mathrm{CE}-i\_\mathrm{EQ}}$$

The equivalent area of each rebar (S_CE-i_EQ_) relative to its distance from the WE (d_i_) is determined from Equation (6) proposed here:

(6) $$S_{\mathrm{CE}-i\_\mathrm{EQ}}=S_{\mathrm{CE}-i}\cdot \frac{d_{\mathrm{MIN}}}{d_{i}}$$

where d_MIN_ is the shortest distance between any of the rebars in the CE and the WE.

**Figure 8:**
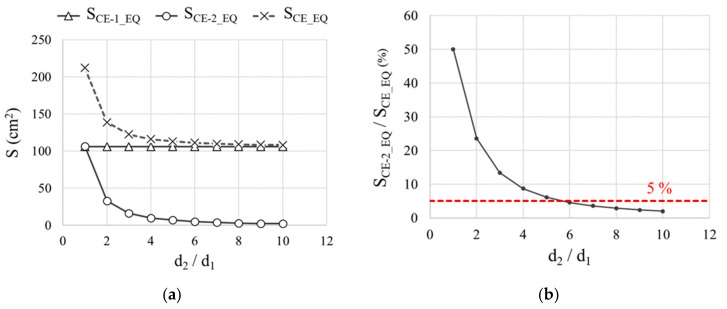
For a two-rebar CE (CE-1 and CE-2) used in Cell 1 (Figure 4) with a deionized water solution with 0 mol·L^−1^ of chlorides: (**a**) CE-1 and CE-2 equivalent areas (S_CE-1_EQ_ and S_CE-2_EQ_) (Equation (6)) together with the equivalent area of the entire CE (S_CE_EQ_) (Equation (5)) for the d_2_/d_1_ ratios studied. (**b**) S_CE-2_EQ_/S_CE-EQ_ vs. d_2_/d_1_.

Figure 8a plots the equivalent areas of CE-1 (S_CE-1_EQ_) and CE-2 (S_CE-2_EQ_) as found with Equation (6) and of the respective CE (S_CE_EQ_) calculated with Equation (5) for the d_2_/d_1_ ratios studied in Cell 1 as depicted in Figure 4. As CE-1 was at the shortest distance from the WE in all cases, so d_MIN_ = d_1_ and consequently S_CE-1_EQ_ = S_CE-1_ for all d_2_/d_1_ ratios. Given that the distance to CE-2 from WE (d_2_) varied, S_CE-2_EQ_ also varied, downward as d_2_ rose. In consequence, the equivalent area of the entire CE (S_CE_EQ_) also drops as the CE-2 distance from WE (d_2_) increases (Figure 8a). When d_2_/d_1_ ≥ 6, S_CE-2_EQ_ affected the value of S_CE_EQ_ by less than 5%, and, therefore (Figure 8b), it could be considered S_CE_EQ_ ≈ S_CE-1_EQ_.

By using S_CE_EQ_ to calculate the equivalent area of the sensor (S_EQ_) (Equation (4)), R_E_S_EQ_ (referred as R_E_S_EQ_-corrected) exhibited more uniform values for all the d_2_/d_1_ ratios in the various cells studied (Figure 7b). That greater uniformity was mirrored in the coefficient of variation (C.V.) values shown in Figure 7c, consistently under less than 1%, i.e., smaller than the 2% to 3% calculated with Equation (3), in which the d_2_/d_1_ ratio was not factored in. Such finely tuned results were found by introducing an empirical constant K into Equation (6) to yield Equation (7):

(7) $$S_{\mathrm{CE}-i\_\mathrm{EQ}}=S_{\mathrm{CE}-i}\cdot {\left(\frac{d_{\mathrm{MIN}}}{d_{i}}\right)}^{K}$$

Further to the iterations conducted, the lowest C.V. values (Figure 7c) were obtained for K = 1.7. The findings analyzed through this stage justified the reliability of S_EQ_ calculated as per Equation (4) to find resistivity from the corrosion sensor measurements.

#### 3.1.2. Electrode Spacing

**Figure 9:**
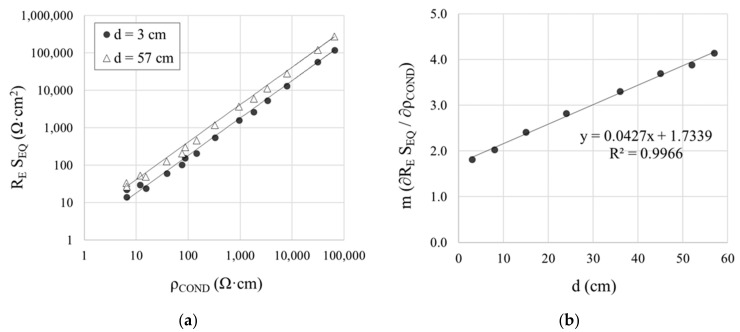
Effect of the distance d between the WE and CE on resistivity measurements in the solutions studied: (**a**) regression line for the relationship between R_E_S_EQ_ and conductivity-meter resistivity (ρ_COND_) for the two extreme distances studied: 3 cm and 52 cm; and (**b**) regression line for the relationship between slope m (∂R_E_S_EQ_/∂ρ) and d.

The curves in Figure 6a show that, as expected, R_E_ varied upward with rising distance (d) between the WE and CE in the cell depicted in Figure 2. That was attested to by the value of R_E_S_EQ_, which, for two clearly divergent distances (3 cm and 52 cm), is graphed in Figure 9a against the conductivity-meter resistivity measurements (ρ_COND_) found in the solutions studied. The R_E_S_EQ_—ρ_COND_ relationship was observed to be linear, for as Table 3 shows, R^2^ was higher than 0.9900 at all distances. The slope m on the R_E_S_EQ_—ρ_COND_ regression line varied upward with rising d, inferring that:

(8) $$R_{E}S_{\mathrm{EQ}}=\rho \cdot m$$

Solving for ρ in Equation (8) to adopt the form of Equation (2) yielded:

(9) $$\rho =\frac{R_{E}S_{\mathrm{EQ}}}{m}$$

The dependence of m (regression line slope) on d (WE-CE distance) graphed in the regression line in Figure 9b is predicted by the following equation:

(10) m=k1·d+k2

where k1 and k2 are constants, respectively related to the slope and the y-intercept for regression line m-d. Substituting Equation (10) into Equation (9) yields:

(11) $$\rho =\frac{R_{E}S_{\mathrm{EQ}}}{k1\cdot d+k2}$$

Equation (11) could consequently be used to obtain the resistivity for the R_E_ value measured between WE and CE, assuming k1 = 0.0427 and k2 = 1.7339. Given that, irrespective of how the sensor is implemented the position of WE relative to CE is known, the value of d is likewise always known.

#### 3.1.3. Electrode Arrangement

As shown in Section 3.1.2, Equation (11) is useful for determining resistivity when the WE and CE are arranged in parallel in the sensor. In practice, however, other setups are possible. Such circumstances were taken into consideration with a study of the seven setups depicted in Figure 5a,b, which envisage some of the most common arrangements for installing sensors in real structures.

**Figure 10:**
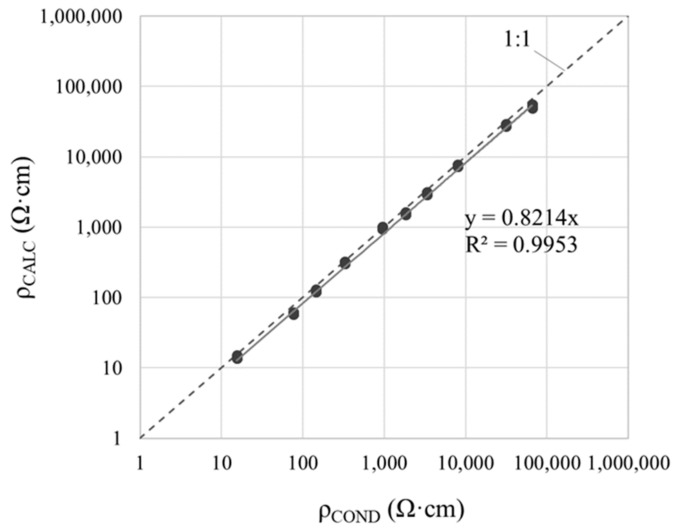
Linear regression between resistivity calculated (ρ_CALC_) with Equation (11) and conductivity-meter measurements (ρ_COND_) (ρ_CALC_ are the mean values listed in Table 4 for the WE-CE setups in Figure 5a,b using solutions B and C).

Table 4 gives the resistivity values determined by applying Equation (11) (ρ_CALC_) with the measurements obtained with the various WE-CE setups. No significant differences were detected between setups or between WE positions I (parallel) and II (45°), with C.V. ≤ 2% in all the solutions studied. The graph in Figure 10 compares the ρ_CALC_ values to the conductivity-meter measurements (ρ_COND_). The R^2^ coefficient was 0.9953, which indicates a good linear correlation between both techniques. According to the slope of the regression line, sensor measurements tend to underestimate ρ by about 18% with respect to the conductivity-meter. That deviation may be deemed acceptable, given the breadth of the resistivity ranges associated with corrosion risk of steel in concrete and the absence of any single criterion on the upper and lower values in each range [6].

### 3.2. Reliability of the PSV-Measured R_E_

The R_E_ measurements discussed in earlier sections were taken with alternating current, referred to in this section as R_E-AC_. Integrating this type of measurement in the INESSCOM sensor system would entail adapting the measuring instrument to alternating current measurements, which would in turn involve more complex electronics and render the system more expensive. Hence the utility of determining R_E_ with the PSV technique presently built into the sensor.

**Figure 11:**
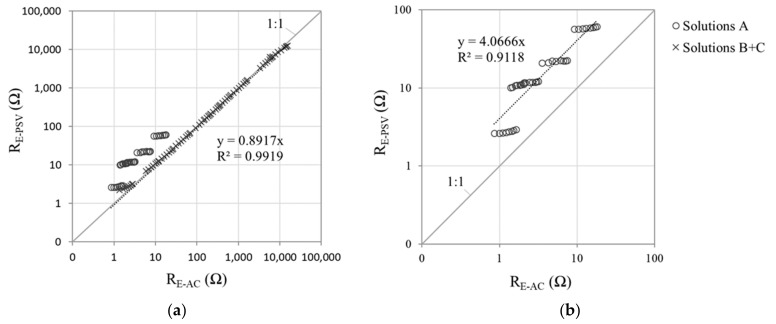
PSV-measured electrical resistance (R_E-PSV_) with the pulse sequence depicted in Figure 1c (sampling time, τ = 100 ms) versus the value found with alternating current (R_E-AC_): (**a**) regression line for solutions in solvents A, B and C, taken jointly; and (**b**) regression for the five solutions bearing solvent A.

As described in Section 2.1, the equivalent circuit used with the PSV technique (Figure 1b) yielded the R_E_ value as per Equation (1), referred to in this section as R_E-PSV_. The regression lines in Figure 11a for all solutions (A, B and C) considered jointly showed a close correlation between R_E-PSV_ and R_E-AC_, with a downward deviation of around 11%. Although that deviation might be deemed acceptable, the values for solvent A (concrete pore solution with different chloride concentrations) were less closely aligned with the general regression trend than those for B and C. The R^2^ value was in fact lower when the R_E-PSV_ and R_E-AC_ values were compared for set A only (Figure 8b) and deviation, upward in this case, was much greater (≈ 400%).

The manner in which R_E_ is found in PSV (R_E-PSV_) lay at the source of that deviation. Equation (1) indicates that R_E-PSV_ was dependent on resistances R_1_ and R_2_ in the model illustrated in Figure 1b. The transient response of the model to the application of a potential step (∆V) at t → 0 depends precisely on these resistances [29]:

(12) $$I_{0}=\Delta V\cdot \left(\frac{1}{R_{1}}+\frac{1}{R_{2}}\right)=\frac{\Delta V}{R_{E-\mathrm{PSV}}}$$

The R_E-PSV_ value was consequently found to be inversely proportional to current intensity at t → 0 (I_0_):

(13) $$R_{E-\mathrm{PSV}}=\frac{\Delta V}{I_{0}}$$

Further to Equation (13), the reliability of R_E-PSV_ depends on whether the value of I_0_ can be accurately recorded with PSV measurements. The pulse sequence used in PSV (Figure 1c) was designed with a 50 s pulse duration and sampling time (τ) of 100 milliseconds. The amount of information recorded under those circumstances was moderate enough for suitable handling, although at the expense of information loss at short times (<0.1 s). That effect was attributed to the deviations in R_E_ observed in Figure 11 for solutions A.

**Figure 12:**
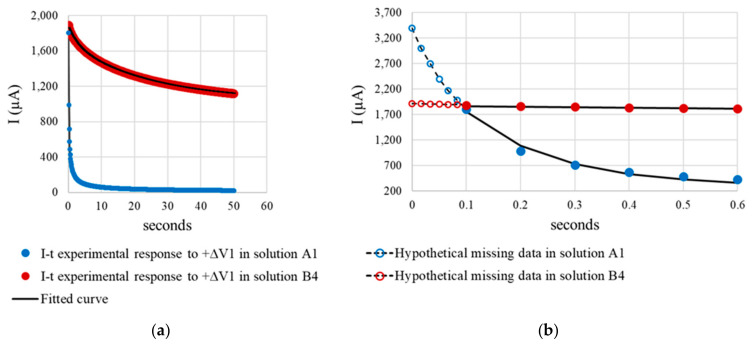
PSV-measured sensor current intensity—time (I-t) response in solutions A1 and B4 for the +∆V1 = 105 mV pulse of the sequence in Figure 1c: (**a**) full I-t response for the 50 s pulse and fitted curve using the model in Figure 1b, and (**b**) response from 0 to 0.6 s, showing the hypothetical part of the curve not recorded prior to 0.1 s.

Figure 12 compares the sensor’s PSV current-time (I-t) response observed for solution A1 to the response for solution B4, both with a very similar R_E-PSV_ value (≈53 Ω to 60 Ω), but at some distance from the R_E-AC_ reference value (14.8 Ω) in A1. For readier comprehension, the comparison focused on the pulse at +∆V1 = 105 mV (Figure 1c), whose fitting parameters for the model in Figure 1b, along with the corrosion current density (i_CORR_) and R_E-PSV_ values recorded during the full PSV test, are given in Table 5.

The shape of the respective I-t curves (Figure 12a) and the R_P_ and i_CORR_ values (Table 5) denoted the existence of two very distinct electrochemical systems. As corrosion kinetics were much slower in A1 (i_CORR_ = 0.672 µA/cm^2^) than in B4 (i_CORR_ = 24.763 µA/cm^2^), capacitors C_1_ and C_2_ are related with the double layer capacitance at the steel-concrete interface (model in Figure 1b) exhibited much lower capacitance in A1 (9412.2 µF and 2225.1 µF) than in B4 (149,367.5 µF and 232,447.5 µF). That explained why current decays abruptly at early times (t < 0.6 s) in A1 while barely varying in B4 (Figure 12b). As Figure 12b shows, then, failing to record the I-t response in A1 for t < 0.1 s may entail greatly underestimating I_0_, whereas in B4 the hypothetical I_0_ value would barely vary. Hence the overestimation (with Equation (13)) of the R_E_ value in A1 (Figure 11b).

**Figure 13:**
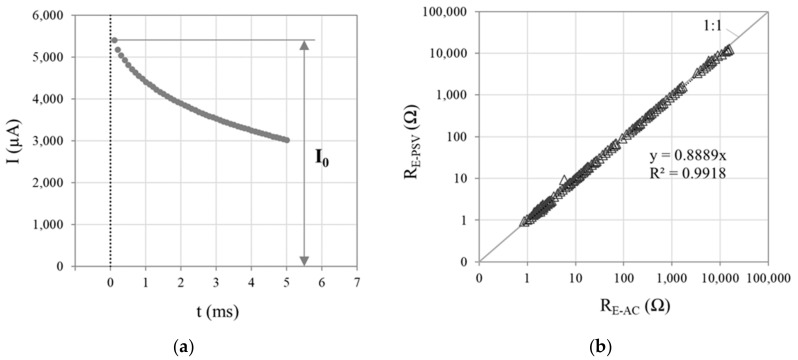
(**a**) Procedure used in the current-time response to a short 50 mV, 5 ms pulse (sampling time τ = 0.1 ms) to find the current at t→0 (I_0_) required to determine R_E-PSV_ with Equation (13); and (**b**) R_E-PSV_ versus R_E-AC_ regression line for solutions bearing solvent A, B or C, considered jointly.

The inference of the foregoing discussion around Figure 12b is that reliable R_E-PSV_ measurement entails the use of short sampling times to suitably record I_0_. Here, as an alternative to the sequence in Figure 1c, a 50 mV, 5 ms pulse was used with sampling time τ = 0.1 ms. As Figure 13a shows, the first point recorded was taken as I_0_, and R_E-PSV_ was determined with Equation (11). With that approach, as the R_E-PSV_ versus R_E-AC_ regression line in Figure 13b shows, no differences in trend were detected for any of the solutions with solvents A, B or C. That procedure consequently corrected the overestimation of R_E_ found for the A solvent solutions using 50 s PSV pulses (Figure 11a), for the deviation between R_E-PSV_ and R_E-AC_ was consistently lower than 12%.

## 4. Discussion

**Figure 14:**
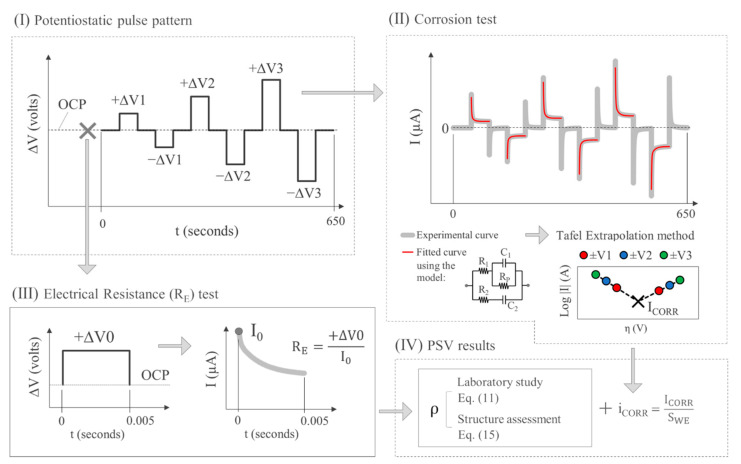
Procedure proposed for resistivity determination by means the PSV technique used in the corrosion sensor described in Section 2.1: (**I**) potentiostatic pulse sequence applied; (**II**) test for determining corrosion current density (i_CORR_) by applying the Tafel extrapolation method from the modelled I-t response; (**III**) test proposed to be included in PSV to determine the R_E_ from the current at t→0 (I_0_) recorded for the I-t response at a +∆V0 = 50 mV, 5 ms pulse; and (**IV**) final PSV results.

The discussion in Section 3.1 and Section 3.2 above resulted in Equation (11), an expression with reliable constant cells to determine resistivity from the R_E_ provided by the INESSCOM embedded sensor. This requires, as identified in Section 3.2, to include a 5 ms, τ = 0.1 ms pulse prior to the original PSV technique used to determine corrosion rate (Figure 13a) Based on these findings, the respective procedure for measuring resistivity based on sensor PSV measurement is illustrated in the flow chart presented in Figure 14.

It should be noted, however, that Equation (11) could be simplified. Given that k1 = 0.0427 and k2 = 1.7339, the product k1·d will practically always be much smaller than k2, whereby the denominator in Equation (11) is not substantially altered by the value of d. Assuming, then, k2 >>> k1·d, Equation (11) can be simplified as follows:

(14) $$\rho =\frac{R_{E}S_{\mathrm{EQ}}}{k2}=\frac{R_{E}S_{\mathrm{EQ}}}{1.7339}$$

In a similar vein, although S_EQ_ can be readily determined from S_WE_ and S_CE_ (Equation (4)) in scaled-down concrete specimens, the S_CE_ value is not always easy to establish in large structures. As the S_CE_ >>> S_WE_ assumption holds in such cases, however, there S_EQ_ may be deemed to be approximately equal to S_WE_. Applying that simplification to Equation (14) yields:

(15) $$\rho =\frac{R_{E}S_{\mathrm{WE}}}{1.7339}$$

Equation (15) is consequently a simplified expression for determining ρ in reinforced concrete structures by using the corrosion sensor described in Section 2.1 and the procedure illustrated in Figure 14.

For a better understanding, Appendix A includes a case study of the embedded-sensor approach proposed for concrete resistivity determination. In addition to the foregoing, it should be noted that the ultimate objective of the embedded sensor approach for resistivity determination is to assess the corrosion risk of the reinforcements arising from the cover concrete properties (porosity, pore fluid chemistry, level of pore saturation, temperature, etc.). Indeed, as is known, there is an inversely proportional relationship between concrete resistivity and reinforcement corrosion rate [5,6].

**Figure 15:**
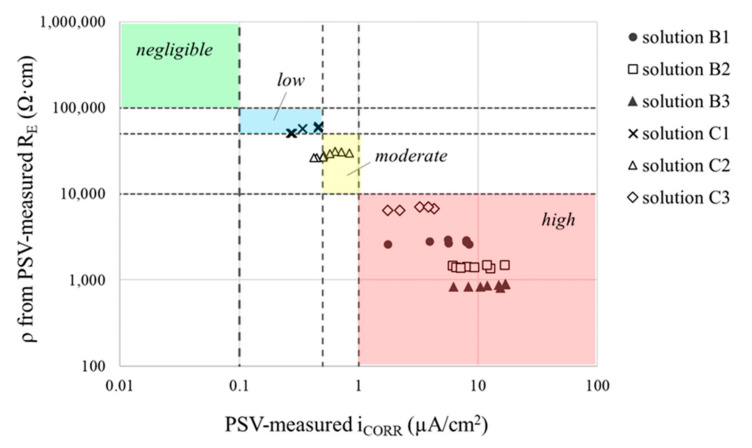
Comparison of resistivity (ρ) and corrosion current density (i_CORR_) obtained by using the procedure in Figure 14 for the different WE-CE distances studied in solutions B1, B2, B3, C1, C2 and C3. The four corrosion risk levels depicted are in accordance with Table 1.

This correlation is confirmed in Figure 15, which compares resistivity (ρ) and corrosion current density (i_CORR_), both measured using the procedure proposed in Figure 14 for the different WE-CE distances studied in the cell of Figure 2. The comparison is focused in solutions B1, B2, B3, C1, C2 and C3, since their resistivity range (Table 2) most closely align with those expected for concrete (Table 1). It is observed an excellent coincidence between the corrosion risk determined from resistivity and from i_CORR_. Therefore, this suggest good reliability of the embedded-sensor approach proposed. However, it should be noted that, as it is known, resistivity is directly affected by temperature [36]. In consequence, temperature should be monitored along with resistivity where temperature cannot be controlled, that is, in field implementations of the INESSCOM sensor. In this way, a temperature correction factor could be determined and applied to reliably determine resistivity. Deeper discussion about this point along with validation of the proposal in concrete specimens will be reported in forthcoming papers.

## 5. Conclusions

We have proposed a reliable expression to determine concrete resistivity from the concrete electrical resistance (R_E_) provided by the embedded corrosion sensor built into the Integrated Network of Sensors for Smart Corrosion Monitoring (INESSCOM) we have developed. This sensor operates using two electrodes: a carbon steel rebar as working electrode (WE) and the reinforcement itself as counter-electrode (CE). The primary goal was to find cell constants valid for any implementation scenario. The conclusions drawn from studying the most significant geometrical features of the sensor in a wide set of calibration solutions are set out below:

(1) The expression ρ = RE·SEQ/(k1·d + k2) is proposed for determining resistivity (ρ) from the RE measured between the WE and CE. Constants are k1 = 0.0427 and k2 = 1.7339. SEQ is the sensor’s equivalent area calculated from the area of WE (SWE) and CE (SCE) as SEQ = SWE·SCE/(SWE + SCE) and d is the WE-CE spacing. The reliability of that calculation is unaffected by how the WE and CE are arranged.
(2) Where the CE comprises n rebars at unequal WE-CE distances, the resulting CE area (SCE_EQ) is the sum of the equivalent area of each of the n rebars (SCE-i-EQ). That calculation assumes SCE-i_EQ = SCE-i · (dMIN/di)K, i.e., SCE-i_EQ is the area of the rebar itself (SCE-i) multiplied by its WE-CE distance (di) divided by the minimum WE-CE distance in the CE (dMIN). An experimental constant, K = 1.7, is required for maximum accuracy.
(3) In electrochemical systems with low corrosion rate and low resistivity, the potential step voltammetry (PSV) technique deployed in the sensor does not provide accurate RE measurements. Consequently, a short sampling time (1 ms) pulse must be added to the original PSV method to provide reliable RE values with a downward deviation of <12% relative to alternating current measurements.
(4) The proposal herein presented for resistivity determination is applicable for RE measurements obtained both using the PSV method (INESSCOM) and alternating current methods. The advantage of using INESSCOM is the possibility of monitoring resistivity along with corrosion rate through a single sensor, which is not usual in structural health monitoring.
(5) The corrosion risk estimations obtained from the resistivity provided by the sensor approach correlated well with corrosion current density determinations in solutions with resistivity close to ordinary concrete values. Deeper discussion about this point along with validation of the proposed resistivity expression using concrete specimens will be reported in forthcoming papers.

## Figures and Tables

**Table 1:** Criteria for assessment of corrosion risk in terms of concrete electrical resistivity (ρ) and corrosion current density (i_CORR_) according to references [8,9,10].

Corrosion Risk	Resistivity (Ω·cm)	i_CORR_ (µA/cm^2^)
Negligible	>100,000	<0.1
Low	50,000–100,000	0.1–0.5
Moderate	10,000–50,000	0.5–1
High	<10,000	>1

**Table 2:** Composition, chloride concentration, pH and resistivity (ρ) of the solutions tested.

Solution ID	Solvent	Cl^−^ (mol·L^−1^)	pH	ρ_COND_ (Ω·cm)
A1	Ca(OH)_2_-saturated tap water	0.0 ^1^	12.54	87.10
A2	Ca(OH)_2_-saturated tap water	0.1 ^1^	12.46	38.71
A3	Ca(OH)_2_-saturated tap water	0.5 ^1^	12.23	11.92
A4	Ca(OH)_2_-saturated tap water	1.0 ^1^	11.98	6.52
A5	Ca(OH)_2_-saturated tap water + 0.22 M NaHCO_3_	1.0 ^1^	8.88	6.60
B1	Tap water	0.0 ^1^	7.50	3347.84
B2	Tap water	0.0015 ^1^	8.37	1830.02
B3	Tap water	0.0032 ^1^	8.54	952.40
B4	Tap water	0.014 ^1^	8.71	326.80
B5	Tap water	0.021 ^1^	8.03	145.77
B6	Tap water	0.1 ^1^	7.80	76.16
B7	Tap water	0.5 ^1^	8.76	15.38
C1	Deionised water	0.0	5.46	65,444.40
C2	Deionised water	0.0	6.62	31,237.83 ^2^
C3	Deionised water	0.00037	6.56	7972.69

^1^ Molar concentration of chloride without considering the contribution of the tap water used in the solvent, which is 0.0005 mol·L^−1^. ^2^ After soaking in the solution for 24 h, resistivity declined due to a sensor corrosion-induced rise in Fe^2+^ and Fe^3+^ concentration.

**Table 3:** Slope (m) and coefficient of determination (R^2^) of the linear regression between the R_E_S_EQ_ and the conductivity-meter resistivity (ρ_COND_) in the solutions at the WE-CE distances (d) studied.

d (cm)	m (∂R_E_S_EQ_/∂ρ_COND_)	R^2^
3	1.6708	0.9977
8	1.8681	0.9991
15	2.2189	0.9983
24	2.5948	0.9955
36	3.0340	0.9909
45	3.3948	0.9918
52	3.5661	0.9917
57	3.8010	0.9917

**Table 4:** Resistivity values found with Equation (11) (ρ_CALC_) using measurements in the seven setups depicted in Figure 5a,b for solutions B and C listed in Table 2, with coefficient of variation (C.V.) (%) for each and the mean ρ_CALC_ intended to mimic the conductivity-meter resistivity measurement (ρ_COND_).

	Setup	WE-CE Position	Sol. B1	Sol. B2	Sol. B3	Sol. B4	Sol. B5	Sol. B6	Sol. B7	Sol. C1	Sol. C2	Sol. C3
ρ_CALC_ (Ω·cm)	1	A	3065.4	1596.6	993.5	317.4	127.5	60.6	14.5	52,099.9	28,606.6	7593.8
		B	3089.1	1603.4	1001.4	321.1	128.1	61.0	14.6	54,026.0	28,607.1	7667.4
	2	A	2947.4	1546.7	963.6	309.0	123.2	58.6	14.0	50,278.2	27,738.6	7354.8
		B	2977.4	1550.8	970.8	318.8	124.2	59.1	14.0	52,281.5	27,819.7	7422.9
	3	A	3016.1	1554.3	968.0	309.4	124.8	59.2	14.3	50,967.1	27,996.0	7425.6
		B	3049.1	1602.7	999.7	320.8	128.5	61.0	14.7	55,025.0	28,714.0	7671.5
	4	A	2882.1	1512.9	943.7	301.3	120.9	57.5	13.7	49,424.0	27,205.1	7210.6
		B	2990.1	1553.5	970.5	311.0	124.3	59.1	14.0	52,940.4	27,854.8	7433.1
	5	A	2978.1	1558.2	973.1	310.0	122.3	59.1	14.1	50,889.9	27,999.4	7423.9
		B	3051.6	1584.6	991.8	317.7	126.8	60.2	14.3	54,092.4	28,527.2	7580.6
	6	A	2917.1	1534.0	958.9	305.3	122.6	58.3	13.7	50,136.5	27,745.9	7330.3
		B	2990.5	1558.5	975.7	312.9	124.7	59.2	14.0	54,252.0	28,167.5	7473.7
	7	A	2937.5	1550.8	967.2	308.8	122.5	58.5	14.0	50,418.2	27,885.3	7404.2
		B	3002.5	1570.7	981.4	314.6	124.9	59.4	14.2	54,355.5	28,384.7	7525.7
	C.V. (%)		2.0	1.7	1.7	1.9	1.9	1.7	2.1	3.6	1.5	1.8
	Mean		2999.6	1562.7	975.7	312.7	124.7	59.3	14.2	52,227.6	28,089.4	7465.6
ρ_COND_ (Ω·cm)			3347.8	1830.0	952.4	326.8	145.8	76.2	15.4	65,444.4	31,237.8	8972.7

**Table 5:** Fitting-curve results for the +∆V1 = 105 mV pulse (Figure 1c) in solutions A1 and B4, showing the value found for the model components (Figure 1b) along with corrosion current density (i_CORR_) and electrical resistance from the original PSV test (R_E-PSV_), and the electrical resistance measured with alternating current (R_E-AC_).

Solution		A1	B4
Curve fitting parameters(for +∆V1)	R_1_ (Ω)	327.2	163.4
	R_2_ (Ω)	72.8	86.0
	C_1_ (µF)	9412.2	149,367.5
	C_2_ (µF)	2225.1	232,447.5
	R_P_ (Ω)	3316.8	15.2
	R_E_ (Ω)	59.5	56.4
Final PSV results	i_CORR_ (µF/cm^2^)	0.672	24.763
	**R_E-PSV_ (Ω)**	**59.3**	**53.6**
**R_E-AC_ (Ω)**		**14.8**	**54.6**

**Figure A1:**
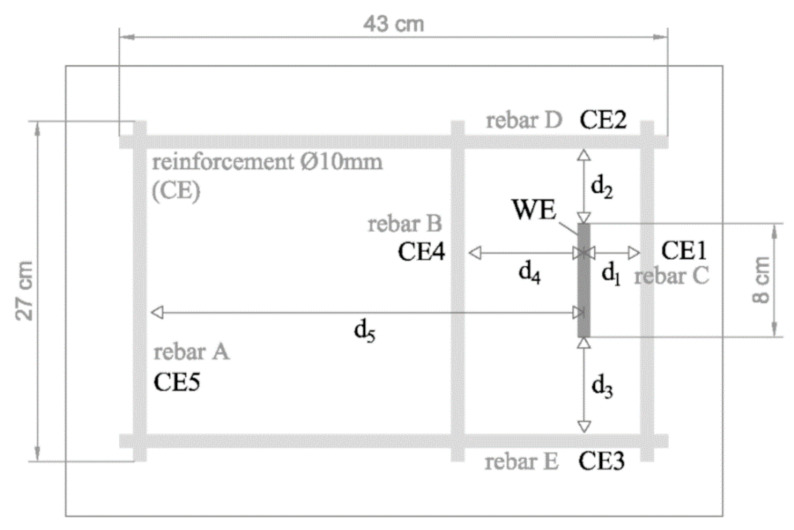
Scheme of the hypothetical reinforced concrete specimen considered in the study case on the application of the embedded-sensor approach proposed in this work to determine the concrete resistivity. The set-up of the corrosion-sensor electrodes (WE and CE) are depicted.

**Figure A2:**
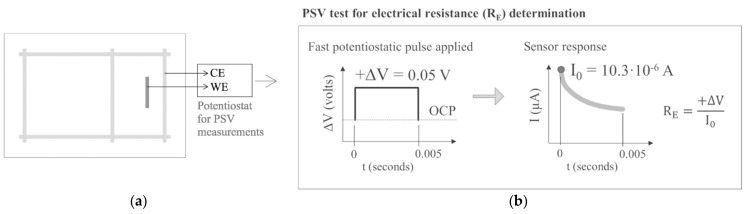
Scheme of the protocol to determine de electrical resistance (R_E_) of concrete by using the PSV technique (implemented in the INESSCOM sensor): (**a**) two-electrode set-up of the corrosion sensor which is connected to the potentiostat used in INESSCOM for PSV measurements; and (**b**) fast potentiostatic pulse (+∆V) applied to polarize the sensor and R_E_ determination from the intensity response of the sensor at t→0 (I_0_).

## Data Availability

The data presented in this study are available on request from the corresponding author. The data are not publicly available due to privacy/ethical restrictions.

## Appendix A

This appendix illustrates a study case on the application of the INESSCOM embedded-sensor approach proposed in this work to determine the concrete resistivity in a hypothetical reinforced concrete specimen. The scheme of the specimen and the sensor set-up is evidenced in Figure A1. The corrosion-sensor electrodes, the working electrode (WE) and the counter-electrode (CE), are embedded in the specimen. The WE is a single short rebar physically isolated from the CE. The CE is the reinforcement itself of the specimen, which consists of five rebars (A, B, C, D and E), each of which (CE-i) is initially numbered according to its distance from the WE (d_i_) (from smaller to greater WE-CE distance); that is CE-1 (at d_1_) = rebar C, CE-2 (at d_2_) = rebar D, CE-3 (at d_3_) = rebar E, CE-4 (at d_4_) = rebar B, CE-5 (at d_5_) = rebar A.

To determine the resistivity in the specimen depicted in Figure A1 using the embedded-sensor approach proposed, the following steps must be taken: (1) Determination of the characteristics of the sensor cell, (2) Measurement of the electrical resistance of concrete (R_E_) and (3) Calculation of the concrete resistivity (ρ). These steps are described in detail in the Appendix A.1, Appendix A.2 and Appendix A.3 that follow.

### Appendix A.1. Determination of the Characteristics of the Sensor Cell

The characteristics of the sensor cell should be collected by using Table A1, Table A2 and Table A3 prior to initiation of the measurements.

**Table A1:** Calculation of the Working Electrode area (S_WE_).

Working Electrode Area (S_WE_)	S_WE_ = 2π·𝜙/2·length = 2π·0.5 cm·8 cm = **25.1 cm^2^**

**Table A2:** Analysis of the characteristics of the Counter-Electrode (CE) and calculation of the Counter-Electrode equivalent area (S_CE_EQ_).

CE ID. According to the WE-CE Distance (from Smaller to Larger)	CE-1	CE-2	CE-3	CE-4	CE-5
Rebar id.	Rebar C	Rebar D	Rebar E	Rebar B	Rebar A
WE-CEi distance (d_i_)	d_1_ = 5 cm	d_2_ = 6.5 cm	d_3_ = 8 cm	d_4_ = 10 cm	d_5_ = 35 cm
CE-i area (S_CE-i_) *	84.8 cm^2^	135.1 cm^2^	135.1 cm^2^	84.8 cm^2^	84.8 cm^2^
d_i_/d_MIN_	1	1.3	1.6	2	7
Rebar to be considered? (d_i_/d_MIN_ ≤ 6)	Yes	Yes	Yes	Yes	No
CE-i equivalent area (S_CE-i_EQ_) $S_{\mathrm{CE}-i\_\mathrm{EQ}}=S_{\mathrm{CE}-i}\cdot {\left(\frac{d_{\mathrm{MIN}}}{d_{i}}\right)}^{1.7}$	84.8 cm^2^	86.5 cm^2^	60.8 cm^2^	26.1 cm^2^	-
**Equivalent total area of the CE** **(S_CE_EQ_)** $S_{\mathrm{CE}\_\mathrm{EQ}}=\overset{n}{\underset{i=1}{\sum }}S_{\mathrm{CE}-i\_\mathrm{EQ}}$	S_CE_EQ_ = S_CE-1_EQ_ + S_CE-2_EQ_ + S_CE-3_EQ_ + S_CE-4_EQ_ S_CE_EQ_ = 84.8 + 86.5 + 60.8 + 26.1 = **258.2 cm^2^**				

* Calculated in the same way as for the S_WE_ in Table A1.

**Table A3:** Calculation of the equivalent area of the Sensor (S_EQ_).

S_CE_EQ_/S_WE_ Ratio	S_CE_EQ_/S_WE_ = 258.2/25.1 = 10.3
If S_CE_EQ_/S_WE_ ≥ 12: $S_{\mathrm{EQ}}≅S_{\mathrm{WE}}$	No -
If S_CE_EQ_/S_WE_ < 12: $S_{\mathrm{EQ}}=\frac{S_{\mathrm{WE}}\cdot S_{\mathrm{CE}\_\mathrm{EQ}}}{S_{\mathrm{WE}}+S_{\mathrm{CE}\_\mathrm{EQ}}}$	Yes $S_{EQ}=\frac{25.1\cdot 258.2}{25.1+258.2}=22.9\;cm^{2}\;$

### Appendix A.2. Measurement of the Electrical Resistance (R_E_) of Concrete

#### Appendix A.2.1. PSV-Measured RE

Figure A2 shows an example of application of the protocol to determine the electrical resistance (R_E_) of concrete by using the PSV technique integrated in the INESSCOM system. The hypothetical R_E_ value obtained is presented in Table A4.

**Table A4:** Calculation of the electrical resistance (R_E_) of concrete according to the protocol depicted in Figure A2.

$R_{E}=\frac{+\Delta V}{I_{0}}=\frac{+0.05\;V}{10.3\cdot 10^{-6}\;A}=4854.4\;\;\Omega$

#### Appendix A.2.2. Alternating Current Methods to Measure R_E_

The R_E_ can be also obtained by using resistance-meter equipment based on alternating current (AC) measurements with a frequency between 50 and 1000 Hz.

### Appendix A.3. Calculation of Concrete Resistivity

The resistivity of concrete (ρ) is determined from the R_E_ measurement with the embedded sensor by applying one of the two equations presented in Table A5.

**Table A5:** Calculation of the resistivity of concrete (ρ) from the R_E_ measurement with the embedded sensor.

Standard Calculation	
$\rho =\frac{R_{E}S_{\mathrm{EQ}}}{k1\cdot d+k2}$	$\rho =\frac{4854.4\;\Omega \cdot 22.9{\;\mathrm{cm}}^{2}}{0.0427\cdot 5\;\mathrm{cm}+1.7339}=57,080\;\Omega \cdot cm$
k1 = 0.0427 k2 = 1.7339 d = d_MIN_	
**Simplified Calculation** **(Use if S_CE_ >>> S_WE_, i.e., in Large Structures)**	
$\rho =\frac{R_{E}S_{\mathrm{WE}}}{1.7339}$	---
